# Kidney Allograft Rupture Following Transplantation: A Scoping Review

**DOI:** 10.1155/joot/9279114

**Published:** 2026-11-03

**Authors:** Hannah Moraa Bosire, Mogamat-Yazied Chothia

**Affiliations:** ^1^ Division of Nephrology Department of Medicine Faculty of Medicine and Health Sciences Stellenbosch University Cape Town South Africa sun.ac.za

**Keywords:** immunosuppression, kidney allograft rupture, kidney transplant, primary graft dysfunction

## Abstract

**Background:**

Kidney transplantation is the preferred therapy for kidney failure; however, complications still occur, among which kidney allograft rupture (KAR) is rare but potentially life‐threatening. This scoping review aimed to map our current understanding of KAR, focusing on clinical presentation, causes of KAR, management strategies and patient and allograft outcomes.

**Methods:**

The review was guided by the Preferred Reporting Items for Systematic Reviews and Meta‐Analyses extension for Scoping Reviews methodology. Eligible studies included case reports, case series and observational studies, including retrospective consecutive series, reporting case‐level or aggregate data on KAR in adults. We searched Medline (PubMed), Scopus and Web of Science Core Collection from database inception to 5 March 2025 and re‐ran all three searches without a study‐design filter between 13 August and 4 September 2026.

**Results:**

A total of 86 articles were included. One hundred and two cases of KAR were analysed at the individual‐patient level, and 34 consecutive series or cohort studies, comprising 367 ruptures among 20 202 transplants, contributed aggregate data; 36 of the 102 individually reported cases were drawn from six of these series and are therefore common to both strands. The mean recipient age was 35 years, and 65% were male. Chronic glomerulonephritis (33%) and hypertension (18%) were the leading reported causes of kidney failure. Acute allograft rejection was the most common precipitating factor for rupture (58%), followed by renal vein thrombosis (14%) and acute tubular necrosis (13%). Clinical presentation was dominated by graft pain or tenderness (88%), a fall in haemoglobin (62%) and hypotension (57%). The allograft was preserved in 49 of 102 cases (48%) and 53 (52%) underwent nephrectomy. Across the 33 single‐centre consecutive series, the pooled incidence of KAR was 1.84% (366/19 922; range between series 0.25%–10.75%), falling from 4.7% in series published before 1990 to 1.5% in those published from 1990 onwards, and 55% of ruptured grafts were preserved. Patient survival was 94%.

**Conclusion:**

KAR is a rare complication of kidney transplantation, occurring in approximately 2% of transplants in consecutive series and most often precipitated by acute allograft rejection. Its reported incidence has fallen roughly threefold since 1990. Just under half of ruptured allografts were preserved and half required nephrectomy. Graft preservation was reported in 62% of the standalone case reports that made up the original version of this review but in only 39% of cases described within consecutive series, indicating that the case‐report literature materially overstates the likelihood of successful salvage. Patient survival was high.

## 1. Introduction

Kidney transplantation is widely recognised as the preferred modality of kidney replacement therapy for patients with kidney failure, as it offers improved survival, enhanced quality of life and reduced long‐term healthcare costs compared to dialysis [1]. Despite advancements in surgical techniques and immunosuppressive therapy, complications such as allograft rupture still do arise [2]. While it is rare, it has severe consequences as it could lead to graft loss or even death and places a strain on healthcare resources [3].

Kidney allograft rupture (KAR) is defined as a superficial or deep tear involving the kidney capsule as well as parenchyma. It frequently occurs along the convex border of the kidney [4]. The incidence of KAR ranges from 0.3% to 9.6% [5]. It was first reported in 1968 by Murray et al. [6] and has since been documented across multiple centres worldwide. It usually occurs within the first 2–3 weeks posttransplant but has been reported to occur as late as 65 months after transplantation in earlier reports [7]. Although its incidence has declined in recent years, possibly due to improved surgical technique and more potent immunosuppressive protocols, KAR continues to present significant clinical challenges.

The pathogenesis is incompletely understood but is strongly associated with acute allograft rejection, which leads to capsular and cortical ischaemia resulting in interstitial oedema and cellular infiltration, creating subcapsular tension that predisposes to tearing [8]. Other reported causes include acute tubular necrosis, renal vein thrombosis, trauma and urinary obstruction [9]. The clinical presentation of KAR is often characterised by acute graft pain, swelling, hypotension and a sudden drop in haemoglobin levels [7]. These hallmark features necessitate immediate investigation, typically with ultrasonography or computed tomography, to confirm the diagnosis and guide prompt management [10].

The therapeutic approach has evolved substantially. While allograft nephrectomy was historically the only management option, the last two decades have seen growing interest in graft salvage strategies. These include the use of haemostatic agents, mesh reinforcement and meticulous suturing to restore structural integrity. Reported salvage success rates range between 40% and 100%, depending on patient selection, timing and surgical expertise [6]. Salvage procedures are increasingly favoured, given the risks associated with nephrectomy, including sensitisation and prolonged waiting times for transplantation. Nevertheless, salvage attempts are not always successful, and nephrectomy remains necessary in cases of uncontrollable bleeding or haemodynamic instability [11].

Despite the growing body of case reports and case series available, a comprehensive synthesis of KAR remains limited. Most publications are retrospective in nature, with small sample sizes and heterogeneous patient populations. As a result, the evidence guiding best practices for prevention, diagnosis and management is fragmented. Therefore, we performed a scoping review to systematically map existing literature; summarise presentation, risk factors and management; document allograft and patient outcomes; and identify research gaps to inform future investigations.

### 1.1. Objectives

The primary objectives of this scoping review were to

1. Map the existing literature on KAR in transplant patients.
2. Identify the typical clinical presentation.
3. Identify the causes/risk factors of KAR.
4. Assess current management strategies.
5. Assess patient and kidney allograft outcomes.

## 2. Materials and Methods

### 2.1. Design and Registration

We conducted the scoping review following the Preferred Reporting Items for Systematic Reviews and Meta‐Analyses extension for Scoping Reviews (PRISMA‐ScR) methodology, and the review is reported in accordance with the PRISMA‐ScR checklist. The review was not prospectively registered. PROSPERO does not accept registrations for scoping reviews, and we did not register a protocol on an alternative platform such as the Open Science Framework, nor was a protocol published in advance.

### 2.2. Eligibility Criteria

We included case reports, case series and observational studies, including retrospective consecutive series and cohort studies, that reported case‐level or aggregate data on the presentation, cause, management or outcome of KAR in adults (≥ 18 years old). KAR was defined as a tear involving both the capsule and the parenchyma of the allograft; reports of rupture confined to the collecting system, and of capsular separation without a parenchymal tear, were therefore not eligible. Studies reporting a consecutive series of transplants were eligible provided that both the number of ruptures and the number of transplants performed were reported, so that an incidence could be derived. Where two reports described the same patients or overlapping cohorts from one centre, the report covering the longer period or the larger denominator was included and the other excluded. We excluded animal studies, studies reporting exclusively on paediatric populations, and narrative reviews that did not report primary data. Eligibility was restricted to articles published in the English language because the review team had neither funding nor institutional support for professional translation.

### 2.3. Information Sources and Search Strategy

We searched Medline (PubMed), Scopus and Web of Science Core Collection from database inception to 5 March 2025; all three databases were searched on 5 March 2025. In that search, the rupture concept was combined with a filter for case reports and case series. Following peer review, we re‐ran all three searches with the design filter removed, so that observational studies of any design could be retrieved. The unfiltered Medline (PubMed) search was run on 13 August 2026 and returned 250 records; the unfiltered Scopus and Web of Science Core Collection searches were run on 4 September 2026 and returned 316 and 203 records, respectively. The unfiltered Medline strategy retrieved every one of the previously included studies that is indexed in that database, confirming that removing the filter did not cost sensitivity, while the re‐run as a whole identified 52 further eligible studies, of which 19 were consecutive series. The original strategies are reproduced below and all six strategies, original and unfiltered, are set out with their dates and yields in Supporting Table S3. Every included study is cited in the reference list; Supporting Table S1 maps each to its reference number and states what it contributed.

### 2.4. Selection of Eligible Research

Records retrieved from all three databases were exported to a reference manager and imported into the Rayyan screening software (https://rayyan.ai/), where duplicate records were identified and removed. Two reviewers (H.M.B., M‐Y.C.) independently screened all titles and abstracts against the eligibility criteria. Full texts of potentially relevant records were then sought and were independently assessed against the same criteria by both reviewers (H.M.B., M‐Y.C.). At both the title/abstract and the full‐text stage, disagreements were resolved by discussion between the two reviewers until consensus was reached; no disagreement required third‐party adjudication. Reasons for exclusion at the full‐text stage were recorded and are reported in Table S2. Reports whose full text could not be obtained were classified as ‘reports not retrieved’; in all but one instance, this was because no full‐text version could be accessed through institutional subscriptions, interlibrary loan or direct contact with the corresponding author, and in the remaining instance because the report was not available in English.

### 2.5. Data Extraction Tool and Data Items

A structured data extraction form was developed a priori in Microsoft Excel by the first reviewer (H.M.B.) and was reviewed and agreed by the second reviewer (M‐Y.C.) before use. Each variable was accompanied by a written operational definition and, where applicable, a prespecified response set, so that extraction was standardised between reviewers. The form was pilot tested independently by both reviewers on a sample of three randomly selected included articles; the results were compared, ambiguous variable definitions were clarified and the form was amended accordingly before extraction of the remaining articles proceeded. No further amendments were required thereafter. All data were subsequently extracted by H.M.B. using the standardised form and were independently verified item by item against the source articles by M‐Y.C. Discrepancies between the extracted data and the source article were resolved by joint re‐examination of the source article and discussion until consensus was reached; no third reviewer was required. Where a variable was not reported in the source article, it was recorded as ‘not reported’ and was not imputed, and the denominator for that variable was reduced accordingly. The data extracted included the type of publication, year of publication, country of origin, demographic data, underlying cause of kidney failure, allograft source, ischaemia times, immunosuppressive medication, clinical presentation, cause/risk factor of KAR, site of rupture, time from transplantation to event, the treatment option applied such as nephrectomy vs. salvage surgery, and patient and allograft outcomes.

### 2.6. Data Synthesis

We provided a descriptive synthesis with tables, figures and narrative summaries. Continuous variables are summarised as mean (SD) or median (IQR) as appropriate, and categorical variables as counts and percentages. Because reporting was incomplete for many variables, the denominator used for each percentage is the number of cases for which that variable was reported, and this denominator is stated throughout. Because the included studies reported data at two different levels, we synthesised them separately. Case reports and case series contributed individual patient records, which were pooled for the case‐level analyses in Tables 1, 2, and 3. Consecutive series contributed aggregate data, summarised separately in Table 4; these were not pooled with the case‐level data, as doing so would double‐count patients and weight the analysis towards the larger series. Six consecutive series also described their patients individually, so 36 cases appear in both strands; these cases are counted once in each analysis and never added across the two. Where a consecutive series reported both the number of ruptures and the number of transplants performed, we calculated the incidence of KAR for that cohort. To examine whether the case‐report literature gives a different picture of outcome from the consecutive series, we compared graft preservation between cases reported as standalone reports and cases reported within a consecutive series. Consistent with scoping review methodology, no meta‐analysis was undertaken, and no formal critical appraisal or risk‐of‐bias assessment of individual sources was performed. All included and cited references were cross‐checked in September 2026 against the Retraction Watch database (https://retractiondatabase.org/) and against PubMed retraction and correction notices. No included or cited article had been retracted, and no published correction was identified that altered the relevance of a cited finding to this review.

**TABLE 1:** Patient and transplant characteristics.

Variable	*n*/*N* (%) or summary data
Recipient age (years), mean ± SD	35 ± 12 (*n* = 70)
Recipient sex	
Male	49/75 (65)
Female	26/75 (35)
Donor age (years), mean ± SD	33 ± 18 (*n* = 40)
Donor sex	
Male	14/26 (54)
Female	12/26 (46)
Cause of kidney failure	
Chronic glomerulonephritis	17/51 (33)
Hypertension	9/51 (18)
Diabetes mellitus	4/51 (8)
Reflux nephropathy	4/51 (8)
Membranous nephropathy	2/51 (4)
Chronic interstitial nephritis	2/51 (4)
FSGS	2/51 (4)
Chronic pyelonephritis	3/51 (6)
CAKUT	2/51 (4)
ANCA vasculitis	1/51 (2)
Transplant characteristics	
Graft source	
Living‐related	19/94 (20)
Deceased donor	71/94 (76)
Living‐unrelated	4/94 (4)
HLA mismatch	
0	3/43 (7)
1	2/43 (5)
2	9/43 (21)
3	16/43 (37)
4	5/43 (12)
5	5/43 (12)
6	3/43 (7)
Ischaemia times	
Cold, mean ± SD	15 h 36 min ± 10 h 44 min (*n* = 19)
Warm, mean ± SD	18.1 min ± 15.5 min (*n* = 11)
Immunosuppressive drugs	
Prednisone	56/63 (89)
Azathioprine	45/65 (69)
Cyclosporin	26/66 (39)
Anti‐thymocyte globulin	13/46 (28)
Mycophenolate mofetil	16/47 (34)
Basiliximab	6/47 (13)
Methylprednisolone	11/46 (24)
Tacrolimus	12/47 (26)
Sirolimus	1/47 (2)
Everolimus	1/46 (2)

*Note:* The categories listed account for 46 of the 51 cases in which a cause of kidney failure was reported; the remaining five cases had other causes.

Abbreviations: ANCA, anti‐neutrophil cytoplasmic antibodies; CAKUT, congenital abnormalities of the kidney and urinary tract; FSGS, focal segmental glomerulosclerosis; HLA, human leukocyte antigen; SD, standard deviation.

**TABLE 2:** Findings during clinical presentation.

Clinical presentation	*n*/*N* (%)
Allograft pain/tenderness	89/101 (88)
Haemoglobin reduction	39/63 (62)
Hypotension	39/68 (57)
Allograft dysfunction	30/63 (48)
Allograft swelling	26/60 (43)
Haematuria	7/61 (11)

**TABLE 3:** Surgical salvage procedures.

Surgical procedures	*n*/*N* (%)
Suture	25/40 (63)
Mesh reinforcement	12/40 (30)
Surgical glue	8/40 (20)
Haemostatic patch	8/40 (20)
Fascia/muscle	6/40 (15)
Haemostatic matrix	3/40 (8)
Argon beam coagulator	4/40 (10)
Omental patch	3/40 (8)

**TABLE 4:** Consecutive series of kidney transplants reporting the incidence and outcome of kidney allograft rupture.

Study	Country	Period	Transplants	Ruptures	Incidence (%)	Grafts preserved, *n* (%)
Finley and Roberts [12]	USA	20 years to 2000	4418	22	0.50	14 (64)
Han et al. [13]	China	Nov 2002–Nov 2008	1851	26	1.40	26 (100)
Hochleitner et al. [14]	Austria	1974–1999	1811	14	0.77	9 (64)
Shahrokh et al. [9]	Iran	1986–2003	1682	6	0.36	2 (33)
He et al. [15]	China	Apr 1991–Aug 2000	1000	38	3.80	34 (89)
Ramos et al. [16]	Portugal	1983–1999	934	11	1.18	1 (9)
Richardson et al. [17]	United Kingdom	Jan 1975–Jan 1989	791	15	1.90	4 (27)
Oktar et al. [18]	Turkey	1983–2017	789	2	0.25	1 (50)
Busi et al. [19]	Italy	not stated	778	4	0.51	1 (25)
Sánchez de la Nieta et al. [20]	Spain	1990–2001	657	10	1.52	3 (30)
Szenohradszky et al. [2]	Hungary	1979–1998	628	53	8.44	16 (30)
Martínez Mansur et al. [21]	Argentina	1992–2005	492	11	2.24	4 (36)
Susan et al. [22]	USA	Jan 1963–Jan 1977	474	4	0.84	4 (100)
Robertson et al. [23]	Australia	Jul 1983–Dec 1990	384	6	1.56	4 (67)
Oesterwitz et al. [24]	Germany	Mar 1968–Dec 1978	364	22	6.04	12 (55)
Goldman et al. [25]	Belgium	not stated	350	7	2.00	3 (43)
Azar et al. [5]	USA	not stated	331	12	3.63	0 (0)
Dzamic et al. [26]	Serbia	not stated	311	5	1.61	3 (60)
Lord et al. [4]	Australia	to 1972	280	1	0.36	0 (0)
Murty et al. [27]	India	1991–2007	245	1	0.41	0 (0)
Heimbach et al. [28]	Germany	Apr 1982–Jan 1994	238	8	3.36	7 (88)
Fjeldborg and Kim [29]	Denmark	1964–1971	200	7	3.50	2 (29)
Gonnermann et al. [30]	Germany	1976–1982	186	20	10.75	15 (75)
Yadav et al. [31]	India	not stated	152	11	7.24	8 (73)
Zadrozny et al. [32]	Poland	1991–1995	112	8	7.14	3 (38)
Minale et al. [33]	Switzerland	1964–1971	100	6	6.00	6 (100)
Schamm et al. [34]	South Africa	2008–2011	100	1	1.00	0 (0)
Thukral et al. [35]	USA	Nov 1973–Nov 1979	100	3	3.00	3 (100)
Dostal et al. [36]	Germany	1972–1975	95	6	6.32	5 (83)
Dryburgh et al. [37]	USA	Jan 1976–Apr 1977	93	9	9.68	1 (11)
Ghose et al. [38]	USA	1966–1972	71	5	7.04	3 (60)
Prabahar and Soundararajan [39]	India	1995–2007	68	4	5.88	2 (50)
Serrallach et al. [40]	Spain	Jun 1980–Oct 1982	66	5	7.58	5 (100)
Reznicek et al. [41]	Slovakia	not stated	51	4	7.84	0 (0)
Pooled, single‐centre consecutive series (*n* = 33)			**19 922**	**366**	**1.84**	**201 (55)**

*Note:* Series are ordered by the number of transplants performed. Incidence is the number of recipients with a rupture divided by the number of transplants over the same period. Lord (1973) reports a single rupture against a multihospital registry denominator rather than a single‐centre consecutive series and is therefore excluded from the pooled estimate, which covers the remaining 33 series. Minale (1972) recorded seven rupture episodes in six recipients; the incidence is calculated per recipient. Definitions of graft preservation are not uniform between series: some report the graft as preserved if it survived the operation, others only if it was still functioning at 1 year, so the pooled proportion should be read as approximate. Oktar (2020) supersedes Kocak (2005), and Szenohradszky (1999) supersedes Szederkényi (1998); in each pair, the two reports describe the same patients and only the larger is included. Thukral (1982) reports three ruptures in 100 consecutive transplants and also contributes its three cases to the case‐level analysis. Values in bold are pooled totals across the 33 single‐centre consecutive series.

## 3. Results

### 3.1. Selection of Included Research

The results of the search strategy are shown in Figure 1. The original searches of 5 March 2025 identified 523 records, of which 172 were duplicates. Of the 351 titles and abstracts screened, 283 were excluded and full texts were sought for 68 reports. Full text could not be obtained for 33 of these 68 reports (49%): in 32 instances, no full‐text version was accessible through institutional subscriptions, interlibrary loan or direct contact with the authors, and one report was not available in English. Of the 35 reports assessed for eligibility, four were excluded because they did not report sufficient case‐level data, leaving 31 studies from the original search. The unfiltered re‐run of all three databases identified 769 further records (Medline 250, Scopus 316, Web of Science 203). After removal of 341 duplicates, within and between databases and against the records already screened, 428 records were screened and 369 were excluded on title and abstract. Full text was sought for 59 reports and obtained for 58. Six reports were excluded with reasons: two because the entity described was not a tear of both capsule and parenchyma (one calyceal rupture secondary to ureteric stenosis and one late capsular decapsulation), three because they described patients or cohorts already represented by another included report, and one because it was a narrative review without primary data. The re‐run therefore contributed 52 further studies. Separately, we re‐checked the reference list of the manuscript as originally submitted against the eligibility criteria and identified three articles that had been cited there as background but had never been carried into the extraction; all three met the criteria and were included. We included a total of 86 articles: [2–5, 7–45] and [46–88], contributing 102 individual cases of KAR and 34 consecutive series or cohort studies (Tables 4 and S1); six studies contributed to both strands. Reasons for exclusion at the full‐text stage are listed in Table S2.

**FIGURE 1:**
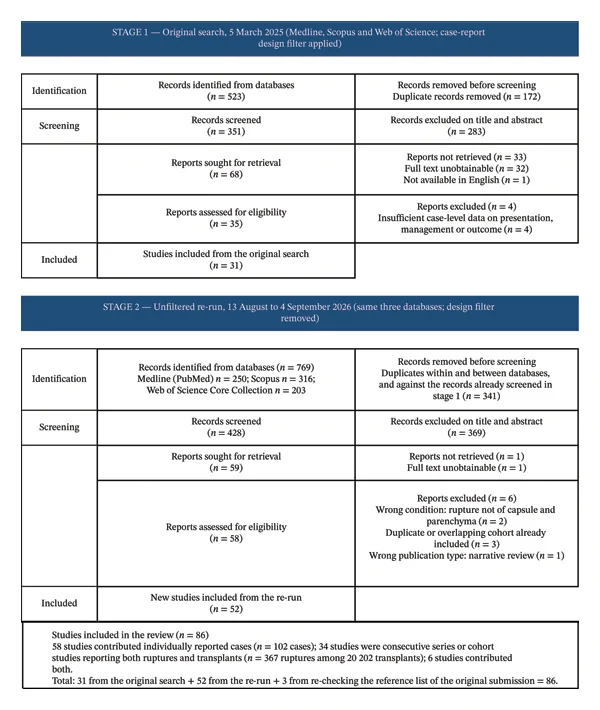
Flow diagram of study selection. Stage 1 reproduces the screening record of the original review. Stage 2 covers the unfiltered re‐run of all three databases undertaken in response to peer review, together with renewed attempts to retrieve the reports that could not be obtained in stage 1. Reasons for exclusion at the full‐text stage are listed in Supporting Table S2.

### 3.2. Characteristics of Included Research

The included articles were published between 1969 and 2024 and originated from 29 countries, most commonly the United States. Of the 86 articles, 58 contributed data on individually described patients and 34 were consecutive series or cohort studies reporting both the number of ruptures and the number of transplants performed; six articles did both.

### 3.3. Synthesis of Results

#### 3.3.1. Summary of Existing Literature

Across the 102 individually reported cases, the mean (SD) recipient age was 35 (12) years [42] among the 70 cases in which it was reported; of the 75 cases reporting sex, 49 were male and 26 female (65% male). Graft source was reported for 94 cases, of which 71 (76%) were from deceased donors, 19 (20%) from living‐related donors and 4 (4%) from living‐unrelated donors.

Among the 51 cases with a reported aetiology of kidney failure, chronic glomerulonephritis (33%) and hypertension (18%) were the most common causes. Data on the type of immunosuppression used were available for 67 patients, although reporting of individual agents was less complete and the denominator therefore differs between drugs. Each patient was on a multidrug immunosuppressive regimen; we therefore analysed the frequency of each drug. The most common drug used was prednisone (56/63, 89%), followed by azathioprine (45/65, 69%) and cyclosporin (26/66, 39%). Anti‐thymocyte globulin (ATG) (13/46, 28%) and basiliximab (6/47, 13%) were used as induction agents in a minority, while tacrolimus (12/47, 26%) and mycophenolate mofetil (16/47, 34%) were used in around a third of cases.

The time to allograft rupture was reported for 77 patients, with a median of 7 (IQR 4–11) days; 63 of the 77 ruptures (82%) occurred within 14 days of transplantation, although the range extended to 19 years. The site of allograft rupture was reported for 42 patients and occurred most frequently along the convex border (19/42, 45%) and at the upper pole (11/42, 26%) (Figure 2).

**FIGURE 2:**
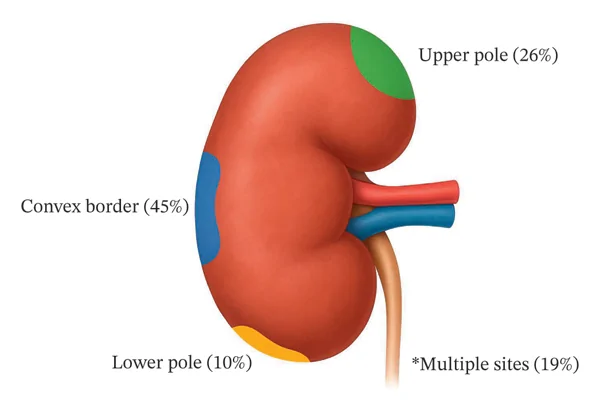
Sites of kidney allograft rupture.

#### 3.3.2. What Was the Typical Clinical Presentation of KAR?

Patients often presented with more than one clinical feature. We analysed the frequency of each among the cases in which that feature was reported. The most frequent presentation was allograft pain or tenderness (89/101, 88%), followed by a fall in haemoglobin (39/63, 62%), hypotension (39/68, 57%), allograft dysfunction (30/63, 48%) and graft swelling (26/60, 43%). Haematuria was uncommon, occurring in 7 of 61 cases (11%).

#### 3.3.3. What Were the Causes/Risk Factors of KAR?

A cause of rupture was documented in 88 of the 102 cases. Acute allograft rejection was the leading cause, reported in 51 patients (58%). Renal vein thrombosis and acute tubular necrosis were the underlying causes in 12 (14%) and 11 (13%) patients, respectively, and trauma in 4 (5%) (Figure 3). The distribution was strongly era‐dependent. In the largest series to report it by immunosuppressive era, rejection accounted for all eight ruptures under azathioprine and prednisolone, whereas renal vein thrombosis was present in all seven ruptures occurring under triple therapy with cyclosporin.

**FIGURE 3:**
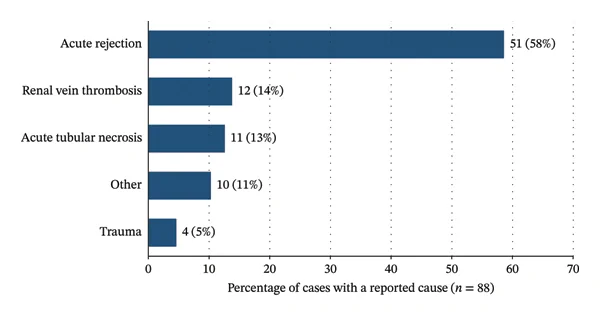
Causes/risk factors of kidney allograft rupture.

#### 3.3.4. What Were the Surgical Salvage Procedures for KAR?

Among the 102 individually reported cases, the allograft was preserved in 49 (48%) and the remaining 53 (52%) underwent allograft nephrectomy, either as the primary procedure or after an attempted repair. The specific technique was reported in 40 of the 49 preserved grafts, across which 69 individual techniques were applied, so more than one technique was used in the majority. The most frequent was direct suture repair (25/40, 63%), followed by mesh or corsetage reinforcement (12/40, 30%) and surgical glue (8/40, 20%). Other techniques are listed in Table 3.

#### 3.3.5. What Were the Reported Patient and Kidney Allograft Outcomes?

Among the 101 patients for whom survival was reported, 95 (94%) survived and there were six deaths. The allograft was preserved in 49 patients (48%) and removed in 53 (52%). The difference between the two literatures was marked: the allograft was preserved in 35 of 66 cases (53%) reported as standalone case reports or small series, but in only 14 of 36 cases (39%) described within a consecutive series. Restricting the comparison to the cases that made up the original, case‐report‐only version of this review, the allograft was preserved in 28 of 45 (62%). Among the 33 single‐centre consecutive series reporting aggregate outcomes, 201 of 366 ruptured grafts (55%) were preserved.

#### 3.3.6. What Was the Incidence of KAR?

Thirty‐four of the included studies reported both the number of ruptures and the number of transplants performed over a defined period, permitting an incidence to be calculated (Table 4). Across the 33 of these that were single‐centre consecutive series, 366 ruptures occurred among 19 922 transplants, a pooled incidence of 1.84%; the remaining study reported a single rupture against a multihospital registry denominator and was therefore not pooled. Incidence varied more than 40‐fold between centres and eras, from 0.25% in a Turkish living‐donor programme to 10.75% in a German series from the late 1970s. The variation was systematic rather than random: series published before 1990 reported a pooled incidence of 4.74% (109/2302), against 1.46% (257/17 620) in series published from 1990 onwards, consistent with a decline in the frequency of KAR as surgical technique and immunosuppression have improved.

## 4. Discussion

In this scoping review, we synthesised available evidence on KAR, a rare but devastating complication following kidney transplantation. In summary, KAR occurred most often in younger recipients, and most donor kidneys were sourced from deceased donors. The leading causes were acute allograft rejection, renal vein thrombosis and acute tubular necrosis. Patient survival was high, at 94% among the individually reported cases. The allograft was preserved in 49 of the 102 individually reported cases (48%) and in 55% of ruptures described in consecutive series (201/366).

The younger age of recipients may have predisposed to a higher risk of allograft rejection because of a more robust immune response. In addition, the median HLA mismatch was three, and studies have consistently reported an association between the degree of HLA mismatch and the risk of acute kidney allograft rejection [89].

A striking feature of this cohort is that the immunosuppressive regimens reported would not be considered contemporary standard of care. Prednisone (89%), azathioprine (69%) and cyclosporin (39%) predominated, whereas tacrolimus and mycophenolate mofetil—the current first‐line maintenance agents—were used in only 26% and 34% of cases, respectively. Lymphocyte‐depleting induction with ATG was given to fewer than one‐third of recipients (28%), despite a median of three HLA mismatches and a predominance of deceased‐donor grafts. It is therefore plausible that comparatively weak immunosuppression contributed directly to the high proportion of ruptures precipitated by acute rejection (58%) observed here. A Cochrane review of 30 randomised trials found that tacrolimus reduced acute rejection at 1 year compared with cyclosporin (RR 0.69, 95% CI 0.60–0.79) and approximately halved steroid‐resistant rejection (RR 0.49, 95% CI 0.37–0.64) [90], and a further Cochrane review found that ATG induction reduced acute rejection by about one‐third relative to no depleting induction (RR 0.63, 95% CI 0.51–0.78) [91]. Directly relevant to KAR, one of the included cohorts identified initial immunosuppressive treatment without ATG as an independent predictor of allograft rupture on multivariable analysis, alongside delayed graft function, recipient age and a peak panel‐reactive antibody titre above 25% [20]. Taken together, these data suggest that a substantial proportion of the ruptures described in this review reflect an era of less potent immunosuppression, and that the rejection‐mediated pathway to rupture may be less prominent under current regimens. This is consistent with the threefold fall in reported incidence between series published before and after 1990 documented above, and it means that the distribution of causes described here should be extrapolated to contemporary practice with caution.

KAR typically developed very early in the postoperative period, with a median time to rupture of 7 days and 82% of ruptures occurring within the first 2 weeks. This early clustering of cases strongly suggests that acute allograft rejection and factors predisposing to delayed graft function are major triggers [7]. In contrast, a few late ruptures were also identified, the latest at 19 years, and these were dominated by trauma and by rupture of a failed graft rather than by rejection, suggesting that late and early rupture are not the same entity. Similar distributions have been noted in historical case series, which consistently describe early rupture as being most closely associated with acute allograft rejection [4, 9]. Just under 90% of patients developed pain or tenderness over the allograft, while haematuria was uncommon at 11%. These findings reinforce that the absence of haematuria does not exclude allograft rupture, and clinicians should maintain a high index of suspicion when unexplained graft pain or haemodynamic instability arises.

Anatomically, ruptures most frequently involved the convex border and the upper pole of the graft, together accounting for 71% of the 42 cases in which the site was reported. These areas are subject to maximal parenchymal tension and vascular strain, which may explain their vulnerability. From a surgical standpoint, this localisation often necessitates reinforcement with mesh or buttressed sutures to achieve durable haemostasis.

Management strategies have evolved considerably over time. In the earlier literature, nephrectomy was often the only treatment option due to concerns about uncontrolled bleeding; in this review, the allograft was preserved in just under half of the individually reported cases. Direct suture repair was the most common technique, while mesh reinforcement and topical haemostatic agents were employed in selected patients. Slightly more than half of the cases ended in nephrectomy, usually because of haemodynamic instability or extensive parenchymal destruction, and in a minority because a technically successful repair was followed by irreversible rejection.

Overall patient survival was high and appears to have improved compared with earlier reports describing higher mortality. This improvement reflects advances in perioperative care and modern surgical techniques. Just under half of the allografts were preserved. Salvage should be attempted whenever the recipient is haemodynamically stable and the graft appears viable, since preserving the allograft avoids the morbidity of returning to dialysis and reduces the sensitisation that complicates future transplantation. The consecutive series make clear, however, that repair does sometimes fail: in one series, three of six grafts repaired with mesh corsetage came to graftectomy for recurrent rupture within 4–15 days; in another, a graft was removed 24 h after repair; and in a third, a graft repaired successfully on day 6 ruptured again on day 39 and was removed. No such failure appears anywhere in the case‐report literature included here, which is itself a measure of how strongly that literature is filtered by publication.

Our findings are consistent with previous literature, which has identified acute allograft rejection as the predominant precipitant of rupture [11, 62]. Ischaemia‐related injury, vascular thrombosis and acute tubular necrosis were less common but clinically relevant contributors. The strong association between acute allograft rejection and rupture highlights the importance of vigilant monitoring of the allograft during the immediate postoperative transplant period with timeous interventions that may prevent progression to rupture.

Re‐rupture of the kidney allograft after successful salvage can occur due to persistent ischaemia or structurally weakened areas within the allograft, as was seen in one of the patients in this review [42]. These compromised segments also predispose the graft to superimposed sepsis, especially when residual haematoma or necrotic tissue is present, highlighting the ongoing vulnerability of salvaged grafts. Close postoperative imaging, infection surveillance and haemodynamic control, therefore, remain essential postsalvage.

Taken together, these results emphasise several important clinical implications. First, transplant teams should maintain a high index of suspicion for rupture in any patient presenting with sudden graft pain, unexplained anaemia, hypotension, or graft swelling in the early postoperative period. Second, early imaging and surgical consultation should be pursued urgently, as timely repair can preserve graft function. Third, salvage should be attempted whenever feasible, provided the patient is haemodynamically stable, as long‐term outcomes are favourable. Finally, these data reinforce the need for institutional protocols that enable rapid mobilisation of surgical, nephrology, radiology and anaesthesia teams when rupture is suspected.

While salvage outcomes have improved substantially, important gaps remain. The evidence base is still dominated by single‐centre experience: the 34 consecutive series identified here span 6 decades and 21 countries, and their reported incidence differs more than 40‐fold, which no single‐centre study can resolve. Multicentre registries are needed to better define incidence in the modern immunosuppression era, to evaluate the durability of allograft salvage over time and to determine whether specific surgical techniques (e.g., mesh versus patch reinforcement) are superior. Further research should also explore the role of preventive strategies, including minimising warm and cold ischaemia times, optimising donor selection and tailoring immunosuppressive regimens in high‐risk patients.

### 4.1. Strengths and Limitations

To the best of our knowledge, this is the first scoping review to report on the clinical features, causes/risk factors and outcomes of KAR. Its strengths include a search of three major databases, run first with and then without a study‐design filter; independent duplicate screening at both the title/abstract and the full‐text stage; independent item‐by‐item verification of all extracted data against the source articles; and a synthesis that keeps individually reported cases and consecutive series separate, which allows the difference between the two literatures to be measured rather than assumed.

Several limitations must be considered when interpreting these findings. The most important is that the individually reported cases come from a literature that is subject to substantial publication and reporting bias. Unsuccessful salvage attempts, perioperative deaths and routine nephrectomies performed for rupture are considerably less likely to be published than novel or successful salvage techniques; several of the included reports were explicitly framed as demonstrations of a new repair method. The clearest signal of this filtering is the gap between the two strands of this review: graft preservation was reported in 62% of the standalone case reports that made up the original version of this review, but in only 39% of cases described within a consecutive series and 55% of ruptures across the series as a whole. Only one of the standalone reports described a repair that failed—Rosario and colleagues [80] repaired all three of their ruptured grafts successfully but lost two to irreversible rejection—whereas the consecutive series describe such failures routinely. The case‐level estimates presented here should therefore be read as best‐case outcomes in selected and reported patients, and the series‐level estimates preferred wherever the two disagree. Selective outcome reporting compounds this, since preserved graft function is more likely to be described, and described in greater detail, than early graft loss. Formal critical appraisal and meta‐analysis were neither performed nor appropriate to a scoping review.

Second, full text could not be obtained for 33 of the 68 reports (49%) sought at the retrieval stage: for 32, no full‐text version was accessible through institutional subscriptions, interlibrary loan or direct contact with the authors, and one was not available in English. This is a substantial proportion of the potentially eligible literature. Because the affected records were disproportionately older publications appearing in journals without digitised archives, the review may under‐represent cases reported in earlier eras, and we cannot exclude the possibility that their outcomes differed systematically from those we were able to include.

Third, eligibility was restricted to English‐language publications because no translation resources were available to the review team. Given the rarity of KAR and the substantial contribution to this literature from centres in continental Europe, Asia and Latin America that frequently publish in local‐language journals, this restriction is a plausible source of language bias and may have led to eligible cases being missed. Any future update of this review should include non‐English reports.

Fourth, three features of the search limit the confidence that can be placed in its completeness. A filter for case reports and case series was applied in all three databases in the original search. That filter demonstrably cost sensitivity: when the searches were re‐run without it, 52 further eligible studies were identified, of which 19 were consecutive series contributing more than 14000 additional transplants. Although Medline offers an indexed publication type for case reports, the Scopus and Web of Science filters relied on the literal phrase appearing in the title, abstract or keywords, which is a less reliable marker still. The unfiltered re‐run was completed between 13 August and 4 September 2026, so reports published after that date are not represented. Finally, the review was not prospectively registered, which limits the assurance that can be given that it was conducted as originally planned.

Fifth, reporting of key variables within the source articles was inconsistent: donor age was reported for 40 of 102 cases, cold and warm ischaemia times for 19 and 11 cases, respectively, HLA mismatch for 43, the aetiology of kidney failure for 51, the immunosuppressive regimen for 67, the site of rupture for 42 and the cause of rupture for 88, so denominators differ between analyses and several estimates rest on small numbers. Patient‐level detail was markedly sparser in the consecutive series than in the standalone case reports, so the case‐level tables are weighted towards the case‐report literature even where the denominator is large. The source reports also did not allow us to distinguish a primary decision to proceed to nephrectomy from a repair that was attempted and subsequently abandoned; we therefore report graft preservation as a proportion of all cases rather than as a success rate among attempted repairs. The definitions of graft preservation used by the consecutive series were not uniform, some reporting the graft as preserved if it survived the operation and others only if it was still functioning at 1 year, so the pooled figure of 55% should be treated as approximate. Finally, many of the included cases originated in earlier immunosuppression eras and may not reflect outcomes achievable under current therapeutic protocols.

## 5. Conclusion

KAR is a rare but serious complication of kidney transplantation, occurring in approximately 2% of transplants across 33 consecutive series and most often precipitated by acute allograft rejection. Its reported incidence has fallen roughly threefold since 1990. Surgical salvage is feasible in many cases and should be attempted whenever clinically appropriate; the allograft was preserved in just under half of the individually reported cases and in 55% of ruptures described in consecutive series. Because graft preservation is reported substantially more often in standalone case reports than in cases described within consecutive series, the case‐report literature should not be used to set expectations of salvage. Early recognition and urgent multidisciplinary management are essential. Multicentre registry studies and prospective cohorts are needed to define the modern incidence of KAR, refine repair strategies and evaluate long‐term outcomes.

## Author Contributions

Mogamat‐Yazied Chothia and Hannah Moraa Bosire conceived and designed the review. Hannah Moraa Bosire developed the search strategies and ran the searches. Hannah Moraa Bosire and Mogamat‐Yazied Chothia independently screened all records at title/abstract and full‐text stage and resolved disagreements by consensus. Hannah Moraa Bosire extracted the data using the prespecified extraction form. Mogamat‐Yazied Chothia independently verified every extracted item against the source articles. Hannah Moraa Bosire drafted the manuscript. Mogamat‐Yazied Chothia supervised the review and critically revised the manuscript for important intellectual content.

## Funding

The authors received no financial support for the research, authorship and/or publication of this article.

## Disclosure

Both authors read and approved the final manuscript and agree to be accountable for all aspects of the work. The research was performed while in the employment of Tygerberg Hospital and Stellenbosch University.

## Ethics Statement

This scoping review synthesised data from previously published studies. It did not involve new research on human participants or animals, and no identifiable individual patient data were accessed. Ethics approval and informed consent were therefore not required.

## Conflicts of Interest

The authors declare no conflicts of interest.

## Supporting Information

Additional supporting information can be found online in the Supporting Information section.

## Supporting information


**Supporting Information** Table S1. Studies included in the review, with full citations. Table S2. Reports excluded at the full‐text stage, with reasons. Table S3. Search strategies, original and unfiltered re‐run. File S1. Completed PRISMA‐ScR checklist.

## Data Availability

The data that support the findings of this study are available in the supporting information of this article.
