# Supplementary material for: Kidney Allograft Rupture Following Transplantation: A Scoping Review
Source: J Transplant. 2026 Nov 3;2026:9279114. doi: 10.1155/joot/9279114 (PMC13729085; doi:10.1155/joot/9279114)
Supplement: Supplementary file 1 [file JOOT-2026-9279114-s001.zip › File_S1_PRISMA-ScR_checklist.docx]

**File S1. Completed PRISMA-ScR checklist**

*Kidney allograft rupture following transplantation: a scoping review*

Checklist items follow Tricco AC, Lillie E, Zarin W, et al. PRISMA Extension for Scoping Reviews (PRISMA-ScR): checklist and explanation. Ann Intern Med. 2018;169(7):467-473. Items 12 and 16 are optional for scoping reviews. Section names refer to the headings used in the main article.

| **#** | **PRISMA-ScR checklist item** | **Reported in this manuscript** |
| --- | --- | --- |
| **TITLE** | | |
| 1 | Title — Identify the report as a scoping review. | Title page. Title reads “Kidney allograft rupture following transplantation: a scoping review”. |
| **ABSTRACT** | | |
| 2 | Structured summary — Provide a structured summary including, as applicable: background, objectives, eligibility criteria, sources of evidence, charting methods, results and conclusions that relate to the review questions and objectives. | Abstract (Background, Methods, Results, Conclusion), page 1. |
| **INTRODUCTION** | | |
| 3 | Rationale — Describe the rationale for the review in the context of what is already known. Explain why the review questions/objectives lend themselves to a scoping review approach. | Introduction, paragraphs 1–4. |
| 4 | Objectives — Provide an explicit statement of the questions and objectives being addressed with reference to their key elements. | Objectives section — five numbered objectives. |
| **METHODS** | | |
| 5 | Protocol and registration — Indicate whether a review protocol exists; state if and where it can be accessed, and if available, provide registration information including the registration number. | Methods, “Design and registration”. The review was not prospectively registered and no protocol was published; this is stated explicitly and repeated as a limitation. |
| 6 | Eligibility criteria — Specify characteristics of the sources of evidence used as eligibility criteria and provide a rationale. | Methods, “Eligibility criteria”. |
| 7 | Information sources — Describe all information sources in the search and date last searched. | Methods, “Information sources and search strategy”. Medline (PubMed), Scopus and Web of Science Core Collection; searched 5 March 2025 and re-run 13 August – 4 September 2026. Supplementary Table S3. |
| 8 | Search — Present the full electronic search strategy for at least one database, including any limits used. | Methods, “Information sources and search strategy”; all six strategies reproduced in full with dates and yields in Supplementary Table S3. |
| 9 | Selection of sources of evidence — State the process for selecting sources of evidence. | Methods, “Selection of eligible research”. Independent duplicate screening at title/abstract and full-text stage; Figure 1. |
| 10 | Data charting process — Describe the methods of charting data, including whether the process was calibrated and whether data were charted independently or in duplicate. | Methods, “Data extraction tool and data items”. Form developed a priori, pilot tested by both reviewers on three articles; all data extracted by HMB and independently verified item by item by MYC. |
| 11 | Data items — List and define all variables for which data were sought and any assumptions and simplifications made. | Methods, “Data extraction tool and data items”. |
| 12 | Critical appraisal of individual sources of evidence — If done, provide a rationale; describe the methods used and how this information was used. | Not performed. Methods, “Data synthesis” states that no formal critical appraisal or risk-of-bias assessment was undertaken, consistent with scoping review methodology. Optional item. |
| 13 | Synthesis of results — Describe the methods of handling and summarising the data. | Methods, “Data synthesis”. Case-level and series-level data synthesised separately; denominators reported per variable; no meta-analysis. |
| **RESULTS** | | |
| 14 | Selection of sources of evidence — Give numbers of sources screened, assessed for eligibility, and included, with reasons for exclusions at each stage, ideally using a flow diagram. | Results, “Selection of included research”; Figure 1 (two-stage flow diagram); Supplementary Table S2 lists full-text exclusions with reasons. |
| 15 | Characteristics of sources of evidence — For each source, present characteristics for which data were charted and provide the citations. | Results, “Characteristics of included research”; Table 4; Supplementary Table S1 (all 86 studies with citations and contribution). |
| 16 | Critical appraisal within sources of evidence — If done, present data on critical appraisal of included sources. | Not applicable — critical appraisal was not performed. Optional item. |
| 17 | Results of individual sources of evidence — For each included source, present the relevant data that were charted that relate to the review questions and objectives. | Tables 1–4; Figures 2 and 3; Supplementary Table S1. |
| 18 | Synthesis of results — Summarise and/or present the charting results as they relate to the review questions and objectives. | Results, “Synthesis of results” and subsequent subsections; Tables 1–4; Figures 2 and 3. |
| **DISCUSSION** | | |
| 19 | Summary of evidence — Summarise the main results, link to the review questions and objectives, and consider the relevance to key groups. | Discussion, paragraphs 1–10. |
| 20 | Limitations — Discuss the limitations of the scoping review process. | Discussion, “Strengths and limitations” — five numbered limitations covering publication and reporting bias, non-retrieval of 33 full texts, English-language restriction, search limitations and non-registration, and incomplete variable reporting. |
| 21 | Conclusions — Provide a general interpretation of the results with respect to the review questions and objectives, as well as potential implications and/or next steps. | Conclusion section. |
| **FUNDING** | | |
| 22 | Funding — Describe sources of funding for the included sources of evidence, as well as sources of funding for the scoping review. Describe the role of the funders. | Funding statement — no financial support was received; research performed while employed by Tygerberg Hospital and Stellenbosch University. |
