# Supplementary material for: Kidney Allograft Rupture Following Transplantation: A Scoping Review
Source: J Transplant. 2026 Nov 3;2026:9279114. doi: 10.1155/joot/9279114 (PMC13729085; doi:10.1155/joot/9279114)
Supplement: Supplementary file 1 [file JOOT-2026-9279114-s001.zip › KAR_supplementary_CLEAN.docx]

**Supplementary material**

*Kidney allograft rupture following transplantation: a scoping review*

**Table S1. Studies included in the review (n = 86)**

*Studies are listed alphabetically by first author. The contribution column states whether a study supplied individually described patients, aggregate data as a consecutive series or cohort, or both. 6 studies did both, so the case and series columns overlap and must not be added together. All 34 studies contributing aggregate data are listed in Table 4 of the main article.*

| **Ref.** | **Study** | **Contribution** | **Cases** | **Transplants** | **Ruptures** | **PMID** |
| --- | --- | --- | --- | --- | --- | --- |
| 12 | Ajao 1984 | Cases | 1 | – | – | – |
| 13 | Akabane 2001 | Cases | 1 | – | – | – |
| 14 | Almarastani 2014 | Cases | 1 | – | – | – |
| 15 | Anderson 1976 | Cases | 1 | – | – | – |
| 8 | Askandarani 2011 | Cases | 1 | – | – | – |
| 16 | Ay 2017 | Cases | 1 | – | – | 26496378 |
| 5 | Azar 1996 | Series | – | 331 | 12 | 8996757 |
| 7 | Baker 2015 | Cases | 1 | – | – | 25685589 |
| 17 | Benjelloun 2005 | Cases | 1 | – | – | 15999620 |
| 18 | Biesenbach 1992 | Cases | 1 | – | – | – |
| 19 | Busi 2004 | Cases and series | 4 | 778 | 4 | 15481704 |
| 20 | Chan 1999 | Cases | 1 | – | – | – |
| 21 | Chopin 1989 | Cases | 3 | – | – | – |
| 22 | Dostal 1976 | Series | – | 95 | 6 | 790058 |
| 23 | Dryburgh 1979 | Cases and series | 8 | 93 | 9 | 378181 |
| 24 | Dzamic 2007† | Series | – | 311 | 5 | – |
| 10 | Favi 2015 | Cases | 1 | – | – | – |
| 25 | Fazeli 2021 | Cases | 1 | – | – | – |
| 26 | Fernandez-Juarez 1998 | Cases | 1 | – | – | 9580227 |
| 27 | Finley 2003 | Series | – | 4 418 | 22 | 12709078 |
| 28 | Fjeldborg 1974 | Series | – | 200 | 7 | 4595810 |
| 29 | Galiano Baena 2013† | Cases | 1 | – | – | – |
| 3 | Gemici 2020 | Cases | 1 | – | – | – |
| 30 | Ghose 1973 | Series | – | 71 | 5 | 4573107 |
| 31 | Godara 2022 | Cases | 1 | – | – | – |
| 32 | Goldman 1978 | Cases | 1 | – | – | – |
| 33 | Goldman 1981 | Series | – | 350 | 7 | 7027550 |
| 34 | Gonnermann 1983 | Series | – | 186 | 20 | 6343051 |
| 35 | Gornacz 1986 | Cases | 1 | – | – | 3530365 |
| 36 | Guleria 1995 | Cases | 1 | – | – | – |
| 37 | Haberal 1974 | Cases | 1 | – | – | 4607455 |
| 38 | Haimov 1971 | Cases | 1 | – | – | – |
| 39 | Han 2012 | Series | – | 1 851 | 26 | 22466908 |
| 40 | He 2003 | Series | – | 1 000 | 38 | 12801330 |
| 41 | Heimbach 1995 | Series | – | 238 | 8 | 7613828 |
| 42 | Hochleitner 2001 | Series | – | 1 811 | 14 | – |
| 43 | Ko 2019 | Cases | 1 | – | – | – |
| 44 | Kohler 1992 | Cases | 1 | – | – | – |
| 45 | Konopa 2024 | Cases | 1 | – | – | – |
| 46 | Kootstra 1974 | Cases | 2 | – | – | 4586368 |
| 47 | Lai 2014 | Cases | 1 | – | – | – |
| 48 | Libertino 1974 | Cases | 1 | – | – | 4591413 |
| 49 | Lord 1970 | Cases | 1 | – | – | 4908951 |
| 4 | Lord 1973 | Cases and series | 1 | 280 | 1 | 4571034 |
| 50 | Mansy 1998 | Cases | 1 | – | – | 9568874 |
| 11 | Maqbool 2022 | Cases | 1 | – | – | – |
| 51 | Martinez Mansur 2006 | Series | – | 492 | 11 | 16903550 |
| 52 | Martinez-Mier 2006 | Cases | 1 | – | – | 16875522 |
| 53 | McCausland 2011 | Cases | 1 | – | – | 21508800 |
| 54 | Millwala 2000 | Cases | 4 | – | – | – |
| 55 | Minale 1972 | Series | – | 100 | 6 | 4552829 |
| 56 | Montali 2022 | Cases | 1 | – | – | 36424228 |
| 57 | Murty 2009 | Series | – | 245 | 1 | 27408183 |
| 58 | Nieto-Rios 2017† | Cases | 1 | – | – | – |
| 59 | Odocha 1991 | Cases | 1 | – | – | 2005656 |
| 60 | Oesterwitz 1980 | Series | – | 364 | 22 | 6995123 |
| 61 | Oktar 2020 | Series | – | 789 | 2 | 32185773 |
| 62 | Ostrovsky 1985 | Cases | 1 | – | – | – |
| 63 | Papadakis 2022 | Cases | 1 | – | – | 34825827 |
| 64 | Philipneri 2000 | Cases | 1 | – | – | – |
| 65 | Picazo 2010† | Cases | 1 | – | – | – |
| 66 | Prabahar 2008 | Series | – | 68 | 4 | 18555124 |
| 67 | Rahatzad 1981 | Cases | 2 | – | – | 7026804 |
| 68 | Ramos 2000 | Series | – | 934 | 11 | 11134721 |
| 69 | Ray 2017 | Cases | 1 | – | – | – |
| 70 | Reznicek 1981 | Series | – | 51 | 4 | 7035388 |
| 71 | Richardson 1990 | Cases and series | 15 | 791 | 15 | 2354344 |
| 72 | Robertson 1992 | Series | – | 384 | 6 | 1586302 |
| 73 | Rosario 1993 | Cases | 3 | – | – | – |
| 74 | Sadasukhi 2023 | Cases | 1 | – | – | – |
| 75 | Said 1994 | Cases | 2 | – | – | – |
| 77 | Schamm 2015† | Series | – | 100 | 1 | – |
| 78 | Schwartz 1986 | Cases | 5 | – | – | – |
| 79 | Serrallach 1985 | Cases and series | 5 | 66 | 5 | 3883006 |
| 9 | Shahrokh 2005 | Series | – | 1 682 | 6 | 16213311 |
| 80 | Shiraishi 2011 | Cases | 1 | – | – | 21846068 |
| 81 | Siedek 1969 | Cases | 1 | – | – | 4391978 |
| 82 | Sin 2014 | Cases | 1 | – | – | – |
| 83 | Susan 1978 | Series | – | 474 | 4 | 341467 |
| 2 | Szenohradszky 1999 | Series | – | 628 | 53 | 10455984 |
| 76 | Sánchez de la Nieta 2004 | Series | – | 657 | 10 | – |
| 84 | Thukral 1982 | Cases and series | 3 | 100 | 3 | – |
| 85 | Vales-Albertos 2006 | Cases | 1 | – | – | – |
| 86 | Yadav 1985 | Series | – | 152 | 11 | 3901474 |
| 87 | Zadrozny 1997 | Series | – | 112 | 8 | 9122940 |
| 88 | Zakir 2003 | Cases | 1 | – | – | – |
|  | **Total** |  | **102** | **20 202** | **367** |  |

*Of the 86 included studies, 58 contributed individually described patients (102 cases in total), 34 contributed aggregate data on 20 202 transplants and 367 ruptures, and 6 contributed both. Those studies appear in both the cases and the series columns, so those two columns must not be added together.*

**Table S2. Reports read in full text and excluded, with reasons (n = 6), and one report not retrieved**

*These are the reports retrieved in full text during the unfiltered re-run and excluded. Reports excluded on title or abstract are counted in Figure 1 but are not listed individually.*

| **Study** | **Title** | **Journal** | **Reason for exclusion** | **Detail** |
| --- | --- | --- | --- | --- |
| Yang 2008 | Late spontaneous kidney graft decapsulation after administration of sirolimus | Transplant Proc | Wrong condition - decapsulation, not rupture | Graft decapsulation with subcapsular haematoma 12 years after transplantation, nine months after conversion from ciclosporin to sirolimus, managed by laparoscopic peritoneal fenestration. No parenchymal laceration is described, so the report does not meet the review definition of allograft rupture as a tear of the renal capsule and parenchyma. |
| Baig 2010 | Post transplant ureteric stenosis causing allograft hydronephrosis and calyceal rupture: salvage side to side ureteroneocystostomy | Saudi J Kidney Dis Transpl | Wrong condition - collecting-system rupture | Calyceal rupture secondary to ureteric stenosis and obstructive hydronephrosis in a 26-year-old woman, salvaged by side-to-side ureteroneocystostomy. This is a forniceal or collecting-system rupture from raised intrapelvic pressure, a different entity from parenchymal allograft rupture. |
| Francis 2018 | Renal allograft rupture | Int J Renal Dis Ther | Wrong publication type - narrative review | Narrative review with no primary patient data and no systematic search. Retains value as a Discussion citation for the reported incidence ranges (2.5-9.6% in early reports, 0.35-2.7% in papers published since 2000) but contributes no cases or denominators. |
| Hegab 2013 | Successful salvage of ruptured living related kidney transplant: a case report | congress abstract | Duplicate publication | Reports the same patient as Almarastani 2014, which is retained: 42-year-old woman, living-related graft, rupture on postoperative day 4, antibody-mediated rejection, graft salvaged, King Abdulaziz Medical City Riyadh. Almarastani is a co-author of both. |
| Kocak 2005 | Urological and surgical complications in 362 consecutive living related donor kidney transplantations | Urol Int | Overlapping cohort | 362 consecutive living-related transplants 1983-2002 at Istanbul University with 2 ruptures. Oktar 2020, which is retained, reports the same unit extended to 789 transplants 1983-2017 and the same 2 ruptures. |
| Szederkenyi 1998 | Rupture of the transplanted kidney: a clinicopathologic study of 37 nephrectomy cases | Transplant Proc | Overlapping cohort | 585 transplants to December 1997 in Szeged with the same 37 nephrectomy cases as Szenohradszky 1999, which is retained because it covers 628 transplants to December 1998 and additionally reports the full rupture count of 53. |
| Nghiem | not retrieved |  | Full text not obtainable | Recorded in the earlier round. Retained here so the full-text exclusion list is complete. |

**Table S3. Search strategies (original and unfiltered re-run)**

*The strategy run in each database on 5 March 2025, and the unfiltered strategy re-run in the same database following peer review.*

| **Database** | **Search** | **Date run** | **Records** | **Search string** |
| --- | --- | --- | --- | --- |
| Medline (PubMed) | Original (design filter applied) | 5 March 2025 | see note | ("renal allograft rupture"[tiab] OR "kidney transplant rupture"[tiab] OR "renal transplant rupture"[tiab] OR "graft rupture"[tiab] OR "transplant rupture"[tiab] OR "kidney graft rupture"[tiab] OR ("kidney transplantation"[MeSH] AND "rupture"[tiab])) AND ("case report"[Publication Type] OR "case reports"[Publication Type] OR "case series"[tiab] OR "case study"[tiab]) |
| Medline (PubMed) | Unfiltered re-run | 13 August 2026 | 250 | (("renal allograft rupture"[tiab] OR "kidney allograft rupture"[tiab] OR "renal transplant rupture"[tiab] OR "kidney transplant rupture"[tiab] OR "renal graft rupture"[tiab] OR "kidney graft rupture"[tiab]) OR (("graft rupture"[tiab] OR "allograft rupture"[tiab] OR "transplant rupture"[tiab] OR "spontaneous rupture"[tiab] OR "rupture"[ti]) AND ("kidney transplantation"[MeSH] OR "kidney transplant*"[tiab] OR "renal transplant*"[tiab] OR "renal allograft*"[tiab] OR "kidney allograft*"[tiab]))) |
| Scopus | Original (design filter applied) | 5 March 2025 | see note | (TITLE-ABS-KEY("renal allograft rupture") OR TITLE-ABS-KEY("kidney transplant rupture") OR TITLE-ABS-KEY("renal transplant rupture") OR TITLE-ABS-KEY("graft rupture") OR TITLE-ABS-KEY("transplant rupture") OR TITLE-ABS-KEY("kidney graft rupture")) AND (TITLE-ABS-KEY("case report") OR TITLE-ABS-KEY("case reports") OR TITLE-ABS-KEY("case series") OR TITLE-ABS-KEY("case study")) |
| Scopus | Unfiltered re-run | 4 September 2026 | 316 | (TITLE-ABS-KEY("renal allograft rupture") OR TITLE-ABS-KEY("kidney allograft rupture") OR TITLE-ABS-KEY("renal transplant rupture") OR TITLE-ABS-KEY("kidney transplant rupture") OR TITLE-ABS-KEY("renal graft rupture") OR TITLE-ABS-KEY("kidney graft rupture")) OR ((TITLE-ABS-KEY("graft rupture") OR TITLE-ABS-KEY("allograft rupture") OR TITLE-ABS-KEY("transplant rupture") OR TITLE-ABS-KEY("spontaneous rupture") OR TITLE("rupture")) AND (TITLE-ABS-KEY("kidney transplant*") OR TITLE-ABS-KEY("renal transplant*") OR TITLE-ABS-KEY("renal allograft*") OR TITLE-ABS-KEY("kidney allograft*"))) |
| Web of Science Core Collection | Original (design filter applied) | 5 March 2025 | see note | TS=("renal allograft rupture" OR "kidney transplant rupture" OR "renal transplant rupture" OR "graft rupture" OR "transplant rupture" OR "kidney graft rupture") AND TS=("case report" OR "case reports" OR "case series" OR "case study") |
| Web of Science Core Collection‡ | Unfiltered re-run | 4 September 2026 | 203 | TS=("renal allograft rupture" OR "kidney allograft rupture" OR "renal transplant rupture" OR "kidney transplant rupture" OR "renal graft rupture" OR "kidney graft rupture") OR ((TS=("graft rupture" OR "allograft rupture" OR "transplant rupture" OR "spontaneous rupture") OR TI=("rupture")) AND TS=("kidney transplant*" OR "renal transplant*" OR "renal allograft*" OR "kidney allograft*")) |

*Both searches were run from database inception with no date or language limit. The original search of 5 March 2025 combined the rupture concept with a filter for case reports and case series; across all three databases it returned 523 records. The re-run removes that design filter, so that observational studies of any design could be retrieved, and adds kidney and renal context terms to the general rupture phrases so that non-renal graft rupture (vascular, tendon, corneal) is not retrieved. The re-run returned 769 records: 250 from Medline, 316 from Scopus and 203 from Web of Science. Field codes differ between platforms: [tiab] and [MeSH] in PubMed, TITLE-ABS-KEY and TITLE in Scopus, TS= and TI= in Web of Science.*

*‡ The Web of Science re-run returned 203 records, but the query string as entered was not retained with the export, and the platform does not record it in the RIS or BibTeX output. The strategy shown is the one drafted for the re-run; the Scopus re-run was confirmed to have been run exactly as drafted. Before submission, re-enter this strategy in Web of Science, confirm it returns 203 records, and correct the string here if it does not.*

*Citations were verified against PubMed records where the study is indexed; the PubMed identifier is given in Table S1 and the full citation in the reference list of the main article. Studies marked † in Table S1 are not indexed in PubMed and their citations were transcribed from the article.*
